# Heart Rate Recovery and Selective Serotonin Reuptake Inhibitors

**DOI:** 10.5935/abc.20160136

**Published:** 2016-09

**Authors:** Levent Cerit

**Affiliations:** Near East University, Nicosia - Cyprus

**Keywords:** Heart Rate, Tobacco Use, Antidepressive Agents

**To the Editor,**

I have read, with great interest, the article entitled "Influence of Smoking Consumption and Nicotine Dependence Degree in Cardiac Autonomic Modulation" by Santos et al.,^1^ recently published in Arquivos Brasileiros de Cardiologia 2016; 106: 510-8. The reseearchers reported that only the intensity of smoking consumption had influences over cardiac autonomic modulation of the evaluated smokers. Smokers with severe smoking consumption intensity presented worse autonomic modulation than moderate ones.^1^

Antidepressant medications are a first-line treatment option for moderate to severe mood and anxiety disorders; however, some studies suggest that long-term use may be associated with an increased risk for cardiovascular disease.^2-4^

Kemp et al.^5^ reported that all users of selective serotonin reuptake inhibitor - except fluoxetine - display alterations in heart rate or heart rate variability (HRV) in comparison to non users. Similarly, users of paroxetine also display small to moderate reductions in HRV relative to users of citalopram, fluoxetine, and sertraline, but not escitalopram.

In this context, it might be beneficial to give more details about medications due to their effect on cardiac autonomic activity.
