# Supplementary material for: Heart Rate Recovery and Selective Serotonin Reuptake Inhibitors
Source: Arq Bras Cardiol. 2016 Sep;107(3):285–6. doi: 10.5935/abc.20160136 (PMC5053200; doi:10.5935/abc.20160136)
Supplement: Related texts

# Reply

**Authors:** Ana Paula Soares dos Santos

Firstly, I would like to thank you for your interest in our manuscript entitled "Influence of Smoking Consumption and Nicotine Dependence Degree in Cardiac Autonomic Modulation" by Santos APS et al., recently published in this magazine.

As described in the Methods section of the study, individuals on medications that influence cardiac autonomic modulation were not included. Antidepressants, which are among said medications, are commonly used by smokers, sometimes as a part of a drug therapy to aid in the cessation of the smoking habit.

Even though participants of the study were part of an Antismoking Awareness and Orientation Program, which consists of the association of cognitive behavioral and drug therapy, they had not yet started the offered treatment, and the cardiac autonomic modulation evaluation was done with smokers who were not abstinent or on specific cessation medication.

Given the above, we can state that, in our study, there was no influence of medications that could alter cardiac autonomic modulation.

**Ana Paula Soares dos Santos**
